# 

**Published:** 1896-09-05

**Authors:** 


### SCRAPS AND GLEANINGS.

During the week ended August 15th 1,091 deaths from cholera in Egypt were recorded.

The workpeople of Huddersfield have increased their subscriptions to the infirmary by £148 this year, the total reaching £1,058.

The managers of the Huddersfield Infirmary have received about half the £8,000 required for building the new wing to the institution.

The Salop Infirmary Hospital Saturday Fund has raised £176 this year, an increase of about 25 per cent. when compared with last year.

Sir W. T. Lewis has decided to contribute £40 a year to the Porth Cottage Hospital to support a bed for sufferers from colliery accidents.

The Devon and Exeter Hospital Committee have decided to lay in the necessary plant for supplying the hospital with electric light, and not to take their supply from the city.

The Lord Lieutenant of the county of Cambridge, who is president of Addenbrooke's Hospital, has given that institution a cheque for £1,000, the estimated cost of the new operating theatre.

The Berwick Hospital and Home for Delicate Girls. established near Dundrum, co. Dublin, under the will of the late Mrs. Harriett Berwick, was formally opened last month by the Archbishop of Dublin.

The number of cases under care in the lunatic asylums of Victoria in 1895 was 4,901, viz., 2,645 males and 2,256 females. Each case cost on an average 9s. 1½d. per week, and the total expenditure amounted to £97,334.

Portsmouth, in the matter of street collections, shows this year a different record to most other towns in England, for £553 was collected in the streets on Hospital Saturday alone, as compared with £487 last year from all sources.

The number of patients treated at the Leeds Public Dispensary shows a slight decrease. The number attending at the dispensary was 31,368, a decrease of 545; and the number visited at their own homes, 2,558, a decrease of 1,019.

The committee of the Royal Cornwall Hospital are endeavouring to raise £800 by October 3rd next, in order to comply with the terms under which a legacy of like amount has been left to them. Up to the end of last month about £520 had been raised.

It is becoming matter for serious consideration how to increase the receipts or to reduce the expenditure at the Belfast Royal Hospital. The expenditure for the eleven months of the current year has exceeded the income by £553, and the hospital had at the end of July to face a debt of nearly £1,200.

The Victoria Jubilee Cottage Hospital at Swaffham has had a fairly prosperous year during the twelve months ended April 30th last. The amount received in annual subscriptions has slightly increased, and the hospital had at the end of the year a balance in hand of £73, against £42 on April 30th, 1895. Fifty patients were treated.

This year, for the first time in its history, the expenditure at the Oswestry Cottage Hospital has exceeded its income. The reasons given for this state of affairs are an increased number of patients, the enlargement of the hospital, the erection of an isolation hospital, and the increase from three to eleven in the nursing staff.

During the year ended June 30th last 668 in and 2,752 out patients were treated at the North Devon Infirmary at Barnstaple. An average of 48 beds were continuously occupied during the year, and each in-patient remained in the hospital on an average for 26 days. The total income amounted to £4,014 (including about £2,000 of extraordinary receipts), and the expenditure to £2,345.

Hull Hospital Sunday in 1895 showed an increase over the previous year of £26, the figures being £784 and £758 respectively. The expenses amounted to £45 or just over 5 per cent. of the total collection. The Church of England contributed 43 per cent.; the Wesleyan Methodists, 26 per cent.; the Primitive Methodists, 8 per cent.; and the remaining denominations 23 per cent. of the amount collected.

The income received during the past year by the Ingham Infirmary, South Shields, was larger than that received in any previous year since its foundation. Owing, however, to the payment of about £210 for new heating apparatus the accounts show a deficit of £44 on the year's working. 8,004 cases were treated, a decrease of 854. The extension scheme, which will provide 48 additional beds, has received the sanction of the governors.

The following statistics circulated by the Bristol Hospital Saturday Fund this year are interesting. Out of a population of 225,000 only 3,500 people regularly subscribe to the hospitals, their contributions amounting to about £8,000, or a little more than one-fourth of the expenditure of the five principal medical charities in Bristol. The orking classes themselves subscribed in 1895 £4,000 towards these charities, which relieved about 85,000 people, or nearly two out of every five of the population of the city.

The Town Moor Management Committee at Newcastle have reported on the application by the Royal Infirmary to hold their exhibition in the Recreation Ground. The committee, while expressing their sympathy with the reason for the exhibition, state that they are met with the difficulty that legally they cannot close the grounds to the public for the six months necessary for the exhibition, and anyone who happened to be disagreeable could successfully oppose its being done. The committee, therefore, shift the responsibility on to other shoulders, and leave the matter in the hands of the council.

The Victoria Infirmary, Lewes, received a large percentage of its income during the year ended Lady Day, 1896, from Hospital Saturday and Sunday. The former collection amounted to over 19 per cent. of the income compared with an average of about 7 per cent. in other provincial general hospitals, and the latter (exclusive of collections in churches other than on Hospital Sunday) to about 11 per cent., as compared with an average of about 7½ per cent. Annual subscriptions amounted to nearly 30 per cent. of the income, about the average in other general hospitals in the provinces and Scotland.

### BOOKS RECEIVED.

Saxon and Co.

"Everybody's Guide to Chess and Draughts." By H. Peachey.

"Everybody's Cycling Law." By Sidney Wright, M.A., and C. W. Browne.

John Wright and Co.

"Galvanism in the Treatment of Neuritis, &c." By Carey Coombs, M.D.

"Our Baby: For Mothers and Nurses." By Mrs. Langton Hewer.

"Rheumatoid Arthritis: Its Pathology, Morbid Anatomy, and Treatment." By Gilbert A. Bannatyne, M.D., F.R.C.P.

Bailliere, Tindall, and Cox.

"Aids to Obstetrics." By Samuel Nall.

"Objects and Limits of Operations for Cancer." By W. Watson Cheyne, M.B., F.R.S., F.R.C.S.

Cassell and Co.

"Food in Health and Disease." By J. Burney Yeo, M.D., F.R.C.P.

Henry Kimpton.

"Obesity: Its Cause and Treatment." By Thos. Dutton.

A. Bonner.

"The Anomalies and Evils of the Medical Administration of India." An address by K. N. Bahadurgi, M.D.

**Periodicals and Pamphlets.**—Knowledge, St. Bartholomew's Hospital Journal, Cassell's Family Magazine, London, Literary Digest, Humanitarian, Medical Week, Zenana, Medical Press, Nursing Notes, Clinical Journal, Australasian Medical Gazette, Woman's Signal, Eclectic Medical Journal, Practitioner, American Journal of Medical Sciences, Medical Bulletin, First Aid, Intercolonial Medical Journal of Australasia, Post Graduate, Public Health, Glasgow Medical Joruna!, Durham College of Medicine Calendar, Charlotte Medical Journal.

### THE STUDENT OF MEDICINE.

### APPOINTMENTS.

E. G. Annis, L.R.C.P.Lond., M.R.C.S.Eng., D.P.H., appointed Medical Officer of Health for the Borough of Huddersfield. H. A. Ballance, M.D., B.S.Lond., F.R.C.S.Eng., appointed House-Surgeon to the Norfolk and Norwich Hospital. H. K. Bean, B.Sc.Edin., M.D.Melb., appointed Government Vaccinator for Wallsend and Plattsburg, N.S.W. J. C. Buckley, M.D.Vict., M.B., Ch.B., appointed Senior Resident Medical Officer to the Nottingham General Hospital. F. J. Burman, L.R.C.P.Edin., M.R.C.S.Eng., appointed Medical Officer of Health for the Wath-upon-Dearne Urban District. J. C. Burton, L.R.C.P.Lond., M.R.C.S.Eng., appointed Resident Medical Officer to the Albany General Hospital, Grahamstown, Cape Colony. J. A. T. Cartwright, M.R.C.S.E., L.S.A., L.M., appointed Medical Officer of Health by the Wigmore Rural District Council. E. Gaylor, L.R.C.P.Edin., L.F.P.S.Glasg., reappointed Medical Officer of Health to the Ripley Urban District Council. J. H. Glover, M.B., appointed Medical Officer for the Bottesford District of the Belfoir Out-Relief Union. S. H. Harrison, M.R.C.S., L.R.C.P., appointed Medical Officer for the North Walsham District of the Smallburgh Union. G. H. S. Hillyar, L.R.C.P., L.R.C.S.Edin, appointed District Medical Officer of the Williton Union. Dr. H. G. Hine, appointed Medical Officer of Health for the Rishworth Urban District. Mr. F. G. W. Hounsell, appointed Medical Officer and Public Vaccinator for the Bugbrooke District of the Northampton Union. C. Lamplough, M.R.C.S., L.R.C.P., appointed Resident Medical Officer to the City of London Hospital for Diseases of the Chest. A. J. Martin, M.B., appointed Medical Officer for the Essington District of the Cannock Union. Dr. McLay, appointed Medical Officer of Health to the Horncastle Urban District Council. R. C. Middlemist, M.R.C.S., L.R.C.P., appointed Medical Officer to the Workhouse of the Stamford Union. J. A. Thompson, M.D., D.P.H., appointed Chief Medical Officer to the Government of New South Wales and President of the Board of Health.

THE STUDENT OF MEDICINE—*continued.*

### VACANCIES.

The following vacancies are announced this week, the amount of salary in each case being given after the name of the institution:—

#### Resident Medical Officers.

City of London Hospital for Diseases of the Chest, Victoria Park, E.—House-Physician. Appointment for six months from October 1st. Salary at the rate of £30 per annum, with board and residence. Applications to J. Storrar Smith, Secretary, by September 9th.

Convalescent Hospital and Sea Bathing Infirmary, Southport.—Resident Medical Officer; unmarried, and not under 47 years of age; doubly qualified. Salary to commence, £150 per annum, with board, lodging, and washing. Applications to the Chairman by September 12th.

Derbyshire Royal Infirmary, Derby.—Clinical Assistant. Appointment for six months. Honorarium, £10 after six months' satisfactory service, with board, residence, and washing. Applications to Walter G. Carnt, Secretary, by September 7th.

Essex and Colchester Hospital.—House Surgeon; doubly qualified. Salary £100 per annum, with board and lodging. Applications to the Committee by September 11th.

London Temperance Hospital. Hampstead Road, N.W.—Assistant Resident Medical Officer; doubly qualified. Appointment for six months. No salary attached to the post, but board, washing, and residence in the hospital provided. Applications to A. W. Bodger, Secretary, by September 16th.

Manchester Southern and Maternity Hospital.—Resident House Surgeon. Honorarium at the rate of £30 per annum, and board. Applications to George William Fox, 53, Princess Road, by September 12th.

Metropolitan Asylums Board.—Assistant Medical Officer at the Fountain Fever Hospital, Lower Tooting, S.W. Salary £160 during the first year, £180 the second year, and £200 during the third and subsequent years of service, with board, lodging, attendance, and washing. Must be unmarried, not exceed 35 years of age, and doubly qualified. Applications, on forms to be obtained at the offices of the Board, Norfolk House, Norfolk Street, Strand, to be sent in by September 7th.

Metropolitan Hospital, Kingsland Road, N.E.—House Physician, House Surgeon, Assistant House Physician, Assistant House Surgeon. Appointments tenable for six months; doubly qualified. The House Physician and House Surgeon will each receive a salary at the rate of £40 a year, the Assistant House Physician and Assistant House Surgeon at the rate of £20 a year. Applications to Charles H. Byers, Secretary, by September 5th.

Royal Free Hospital, Gray's Inn Road, W.C.—Two House Physicians. Appointments for six months. No salary, but board, &c., provided. Applications to the Secretary by September 21st.

Somerset and Bath Lunatic Asylums (Western Joint Asylum), Cotford, near Taunton.—Medical Superintendent. Salary, £450 per annum, with partly furnished house, coals, light, washing, milk, and garden produce. Applications to John Coates, Clerk to the Visiting Committee, Somerset and Bath Asylum, Wells, Somerset, by October 1st.

Victoria Hospital for Sick Children, Queen's Road, Chelsea, S.W.—House-Surgeon; must be F. or M.R.C.S.Eng., and L.S.A. Honorarium of £50 per annum, with board and lodging in the hospital. Appointment for twelve months. Applications to Commander Blount, R.N., Secretary, by September 12th.

#### Non-Resident Medical Officer.

Owens College, Manchester.—Senior and Junior Demonstrator in Physiology. The stipends £150 rising to £200, and £100 rising to £150 respectively. Applications to S. Chaffers, Registrar, by September 22nd.

### PASS LISTS.

#### Society of Apothecaries of London.

The following candidates passed in:—

Surgery.—G. B. Brown, Guy's; A. N. Clemenger, St. George's; O. W. Gange, University College; G. R. B. Gandoin, Madras; H. A. J. Gidney, Calcutta; H. R. Hurry, Melbourne; J. M. Martin, Cambridge and University College; E. A. Quirke, Birmingham; J. K. H. Smyth, St. Mary's; P. R. Wallis, University College.

Medicine, Forensic Medicine, and Midwifery.—W. P. Bean, Leeds; H. A. Belbin, Sheffield and University College; J. O. Garland, Guy's; C. J. Macdonald, St. Bartholomew's; J. M. Martin, Cambridge and University College.

Medicine and Forensic Medicine.—F. M. Cooper (Intermediate), Royal Free; F. E. Saunders, Cambridge and St. Thomas's.

Medicine and Midwifery.—V. A. Settle, Middlesex.

Medicine.—R. N. de Beauvais, Aberdeen and St. Mary's; F. R. M. Heggs, Birmingham; H. Herbert, London; W. Latham, Manchester; J. Ponsonby, Guy's; C. H. Thomas, London.

Forensic Medicine and Midwifery.—G. B. Brown, Guy's.

Forensic Medicine.—T. J. Bokenham, St. Bartholomew's; G. R. B. Gandoin, Madras; W. Lloyd, London; W. A. Pierce, Liverpool.

Midwifery.—P. C. Higgins, Guy's.

The diploma of the Society was granted to the following candidates: Messrs. Bean, Bokenham, Clemenger, de Beauvais, Gange, Higgins, Hurry, Martin, Saunders, Settle, Thomas, and Wallis.

